# Editorial: Global excellence in oncology: Asia and Australia 2021

**DOI:** 10.3389/fonc.2022.980419

**Published:** 2022-08-10

**Authors:** Kun Zhang

**Affiliations:** Central Laboratory, Shanghai Tenth People’s Hospital, Tongji University School of Medicine, Shanghai, China

**Keywords:** oncology, Australia, Asia, diagnosis, therapy

Cancer is one of the most challenging healthcare issues worldwide. In 2021 we called for submissions on the topic of cancer prevalence in Asia and Australia. We received 13 submissions, contributed by 89 authors, which have already had 6,583 views at the time of writing.

Park et al., in their analysis of antitumor medicine consumption in South Korea in recent decades, reported that, among breast, colorectal, liver, lung, gastric, and prostate cancer patients, breast cancer patients were the greatest consumers of antitumor medicine. This analysis provides data-based evidence for policymakers seeking to make cost efficiency improvements. Park et al. reported on postpartum breast cancer, analyzing the incidence rate, related factors, and prognosis according to the timing of breast cancer. Wang et al. analyzed the 5-year relative survival (RS) rate among liver cancer patients in a small city located southeast China, which was reported as 32.4% during the period 2014–2018. To investigate the association between bone cancer (BC) mortality and sex, age, and premature death, Ma et al. conducted population-based epidemiological research for BC mortality in Pudong, Shanghai, China. It was observed that a decrease in BC mortality accompanied the urbanization of the city, and there were clear disparities according to sex and age. Zheng et al. used The Cancer Proteome Atlas and bioinformatics technology to build a predictive model for the prognosis of stomach adenocarcinoma (STAD). The result was a prognostic model consisting of three proteins [collagen VI, cluster of differentiation 20 (CD20), and TP53-inducible glycolysis and apoptosis regulator (TIGAR) ] that can predict 3-year overall survival rates. The study may set the tone for further research on STAD. As for colorectal cancer, Li et al. have developed a machine learning-based CT radiomics model to predict metachronous liver metastasis (MLM) in patients with colorectal cancer. A fusion model combining radiomics and clinical features seems to be a valuable model for predicting MLM. Seeking to improve pelvic radiotherapy, O’Connor et al. compared several methods of synthetic computed tomography (sCT) for MRI-only planning in radiation therapy, demonstrating that bulk density assignment, tissue class segmentation, hybrid atlas, and deep learning sCT generation methods can greatly expand the accessibility of MRI-only planning in radiation therapy.

In research on tumors, Wang et al. found that phospholipase D2 (PLD2) plays a critical role in cancer cell motility and migration and other pathophysiological processes, suggesting that it could be a new therapeutic target for cancer treatment. Xi et al. have reported that microbubbles ultrasonic cavitation can lower the interstitial fluid pressure (IFP) in tumor tissues, leading to an accumulation of sonosensitizers, increasing the therapeutic effects of sonodynamic therapy (SDT). In non-small cell lung cancer (NSCLC), Wang et al. found that profilin 1 (PFN1) oversecretion increased microvesicle (MV) secretion through the rho kinase (ROCK)/phosphorylated myosin light chain (p-MLC) pathway, promoting NSCLC metastasis. By that means, PFN1 is a potential therapeutic target for NSCLC metastasis. Moreover, by reducing the secretion of MVs, it may be possible to partially reverse PFN1 overexpression-induced NSCLC cell migration. This study suggests a potential new approach in NSCLC metastasis treatment.

Regarding diagnostic imaging of the abdomen, Zhang et al. have discussed several intravenous contrast media (CM) for liver imaging according to biobehavioral features, such as an internal distribution and metabolization pathway, and acknowledge the potential value of a ‘summarized navigating map’ of CMs for common use in the clinic. Beyond the wash-in and wash-out data provided by common blood pool contrast agents, additional Kupffer phase data could be provided by Sonazoid^®^, GE Healthcare, Chicago USA, a micrometer-scaled microbubble with inert gas enveloped inside as an ultrasound contrast agent (UCA). Reticuloendothelial system-specific and hepatocyte-selective contrast agents can result in images that are more informative in diagnostic MRI. Moreover, certain molecular imaging agents, including immune molecular probes, stimulus-responsive/microenvironment-dependent contrast agents, and scale-dependent particles, constitute a blueprint for future liver imaging. Hu et al. discuss the clinical characterizations of pancreatic cystic neoplasms (PCNs), including intraductal papillary mucinous neoplasms (IPMNs), mucinous cystic neoplasms (MCNs), solid pseudopapillary neoplasms (SPNs), and serous cystic neoplasms (SCNs), associated diagnostic imaging and precision imaging for radiomics, and emerging techniques such as endoscopic ultrasound (EUS) with fine-needle aspiration (FNA) and molecular markers that compensate for the limitations posed by cytology and tumor markers.

Readers will certainly benefit from the above research.

## Author contributions

The author confirms being the sole contributor of this work and has approved it for publication.

## Conflict of interest

The author declares that the research was conducted in the absence of any commercial or financial relationships that could be construed as a potential conflict of interest.

## Publisher’s note

All claims expressed in this article are solely those of the authors and do not necessarily represent those of their affiliated organizations, or those of the publisher, the editors and the reviewers. Any product that may be evaluated in this article, or claim that may be made by its manufacturer, is not guaranteed or endorsed by the publisher.

